# The impact of Nrf2/HO-1, PD-L1/Bax/Bcl-2 and TNF-α/Il-6 signaling pathways in the ameliorative role of cordycepin against acrylamide-induced cardiac toxicity in rats

**DOI:** 10.1038/s41598-025-08525-x

**Published:** 2025-07-02

**Authors:** Ahmed A. Sedik, Soha A. Hassan, Mona S. O. Gouida, Eman Khalifa

**Affiliations:** 1 Pharmacology Department, Medical Research and Clinical Studies Institute, National Research Centre, Cairo, 12622 Egypt; 2 Department of Biotechnology, Faculty of Applied Health Sciences Technology, October 6 University, October 6 City, Egypt; 3 Genetics Unit, Faculty of Medicine, Children Hospital, Mansoura University, Mansoura, 35516 Egypt; 4 Medical Laboratories Department, Faculty of Applied Health Sciences Technology, Nile Valley University, Fayoum, 63518 Egypt

**Keywords:** Acrylamide, Cardiac toxicity, Cordycepin, Antioxidant, Anti-inflammatory, Environmental sciences, Tumour-necrosis factors

## Abstract

Acrylamide (ACR) is a hazardous and possibly carcinogenic chemical compound that humans are constantly exposed to in their daily life. ACR passively affect many organ systems, including the cardiovascular system. Cordycepin (Cor) is a bioactive molecule derived from various species in the fungal kingdom, especially the genera Cordyceps and Ophiocordyceps. Cordycepin is known for its large-scale pharmacological activities. This study was conducted to evaluate the potential therapeutic effect of Cor on ACR-induced cardiotoxicity. Thirty-two Sprague‒Dawley rats were randomly divided into four groups (8 rats/group). Group I represented the control group, Group II received ACR (20 mg/kg/day) orally for 27 days, and Groups III and IV represented the control group. Rats received ACR (20 mg/kg) orally concurrently with Cor (10 and 20 mg/kg, b.w.), respectively, for 27 days. Serum cardiac biomarkers, such as cardiac troponin I (cTnI), creatine kinase (CK)-MB and lactate dehydrogenase (LDH), were evaluated. In addition, the antioxidant status and inflammatory response to ACR were evaluated. Moreover, detailed histopathological and ultrastructural observations were conducted. Our findings revealed that rats subjected to ACR presented increased levels of serum cardiac biomarkers, tumor necrosis factor-alpha (TNF-α), interleukin-6 (Il-6), malondialdehyde (MDA), annexin V, and Bax values, and programmed death ligand-1 (PD-L1) and decreased activities of nuclear factor erythroid 2-related factor 2 (Nrf-2) and heme oxygenase-1 (HO-1) and reduced glutathione (GSH), superoxide dismutase (SOD) and Bcl-2 expression. Conversely, the administration of Cor to rats treated with ACR in a dose-dependent manner succeeded in restoring serum cardiac biomarkers, modulated redox homeostasis and reduced inflammatory and apoptotic biomarkers. Consequently, a marked improvement was observed in the histopathological and ultrastructural images of the heart. In conclusion, Cor provides cardiac protection by inhibiting ACR-induced Nrf2/HO-1 and Bax/Bcl2 signaling failure, hence maintaining heart function. These findings suggest that Cor could be a promising treatment candidate for reducing ACR-induced cardiac damage.

## Introduction

Cardiovascular diseases (CVDs) are the leading cause of death worldwide, with an average of 17.9 million people affected globally. Cardiometabolic, behavioral, environmental, and social risk factors are major drivers of CVD^1^**.** Acrylamide (ACR) is an environmentally hazardous chemical that is regularly used in various industries and is usually found in foods high in carbohydrates that are cooked at high temperatures^2^. The major source of exposure is through food, cigarette smoke, and occupational exposure^3^**.** ACR has been linked to several health challenges, such as cardiotoxicity, neurotoxicity, hepatotoxicity and infertility^4,5^**.** Furthermore, long-term exposure to ACR may result in endemic health consequences, including an increased risk of developing certain cancers^6^.

Numerous human and animal studies have been performed to determine the specific underlying mechanism of ACR toxicity. Accumulating evidence suggests that ACR toxicity is caused primarily by an oxidative stress insult in which ACR is converted by cytochrome P450 enzymes to a more reactive metabolite, glycidamide (GA), which conjugates with glutathione (GSH), depleting it and increasing reactive oxygen species (ROS) generation, aggravating the oxidative stress process^7–9^. Among the numerous mentioned toxic effects of ACR, cardiac toxicity is a major public health concern.

A potential therapeutic target of cardiac toxicity is the Nrf2 pathway. This transcription factor regulates gene expression in antioxidant defense and detoxification via an antioxidant response element (ARE) that activates heme oxygenase-1 (HO-1)^10^. Superoxide dismutase (SOD) and catalase are two antioxidant enzymes produced as sequelae of Nrf2 activation that can scavenge ROS and protect against oxidative stress when Nrf2 is activated^11^. Inflammatory cytokines can be produced, including tumor necrosis factor-alpha (TNF-alpha) and interleukin-6 (IL-6)^12^. Identifying treatment targets capable of reducing their effects is even more important, given the intricacy of the biochemical mechanisms underlying ACR-induced cardiac toxicity.

Cordycepin is a bioactive molecule known as 3’-deoxyadenosine, which is a nucleoside analogue derived from various species in the fungal kingdom, mainly the two genera Cordyceps (e.g., the species Cordyceps militaris) and Ophiocordyceps (e.g., the species Ophiocordyceps sinensis)^13^. Cordycepin can be synthesized and produced by many biotechnological and industrial processes^14^. The great similarity between Cordycepin and adenosine enables this unique molecule to play a bioactive therapeutic role, as it effectively acts as an antiviral (also in viral myocarditis), antifungal, antibacterial, antiprotozoal, antimicrobial, anti-inflammatory, antioxidant (immunomodulatory/immunoregulatory, antileukemic antitumor/anticancer antiproliferative, antifibrotic, and antimetastatic agent^15^**.** Interestingly, Cordycepin acts as a cardiovascular protection molecule through its role as an antihyperlipidemic antiarrhythmic, antitachycardic, coronary vasodilator, antihypertensive, angiogenic, antiatherosclerotic, antiplatelet aggregation, antithrombotic/thrombolytic/fibrinolytic, anti-ischemic and antistroke agent^16,17^. This study aimed to evaluate the potential therapeutic role of cordycepin in treating ACR-induced cardiotoxicity and to investigate the mechanisms involved in cardiovascular protection.

## Materials and methods

### Chemicals

Acrylamide (ACR) was purchased from Sigma‒Aldrich, USA (CAS number: 79–06-1). Cordycepin (Cor) was obtained from Merck (CAS NO: 73–03-0), Germany. ACR and Cor were freshly prepared in saline.

### Animals

Adult male Sprague dewely male rats weighing 150–170 g were obtained from the animal breeding unit of the National Research Centre (NRC, Giza, Egypt). The rats were acclimatized for one week before any experiments were carried out and were kept under standard environmental conditions (temperature, 23 ± 2°C; humidity, 50%; light system, 12:12 dark/light system). A commercial diet and water were provided ad libitum*.* The experimental study was carried out following the relevant guidelines and regulations approved by the Medical Research Ethics Committee (MREC) of the National Research Centre (approval number: 01451023), and all methods were conducted in accordance with the relevant scientific guidelines and regulations. The current study is reported in accordance with the ARRIVE guidelines.

### Experimental design

Thirty-two male Sprague‒Dawley rats were randomly divided into four groups (8 rats/group). Group I: Rats were orally administered saline for 27 days. Group II: Rats received ACR (20 mg/kg/day) orally for 27 days^18,19^. Groups III and IV: Rats received ACR (20 mg/kg) orally concurrently with Cor (10 and 20 mg/kg, b.w.), respectively, for 27 days^19,20^**.** After the last dose of ACR or Cor, blood samples were collected from the retro-orbital plexus, anaesthetized with ketamine (100 mg/kg) and xylazine (10 mg/kg), allowed to clot, and centrifuged at 4000 rpm for 10 min to evaluate the serum levels of the following cardiac injury indices: cardiac troponin I (cTnI), creatine kinase (CK)-MB and lactate dehydrogenase (LDH). Next, the rats were euthanized with CO₂, and their hearts were excised and weighed. A small portion was washed in cold phosphate-buffered saline (PBS), and parts of the left ventricle were kept frozen in liquid nitrogen. Other samples were homogenized in 10 mM ice-cold Tris–HCl buffer (pH 7.4) and centrifuged at 6000 rpm for 10 min, and the clear supernatant was used to evaluate the expression levels of malondialdehyde (MDA), reduced glutathione (GSH), and superoxide dismutase (SOD) and to evaluate the expression levels of PD-L1, Bcl-2, Bax, Il-6 and annexin V. Other samples from the heart were fixed in 10% neutral buffered formalin to evaluate the histopathology and ultrastructure of the heart.

### Evaluation of serum levels of cTnI, CK-MB and LDH

Serum cTnI was assayed via an ELISA kit (MyBiosource, San Diego, CA, USA), and CK-MB and LDH levels were determined via reagent kits purchased from Spinreact (Girona, Spain) following the manufacturers’ instructions.

### Evaluation of the levels of MDA, GSH and SOD in cardiac tissue

The cardiac value of MDA was assayed as previously described^21^. Briefly, the heart tissue homogenate was mixed with TBA, SDS, and acetate buffer and heated in a boiling water bath for 60 min. After cooling, n-butanol was added to the mixture, which was subsequently centrifuged, and the absorbance of the organic layer was measured at 532 nm. GSH was determined according to the method of Ellman^22^. This assay is based on the reaction of GSH with 5, 50-dithio-bis (2-nitrobenzoic acid) and measurement of the absorbance of the yellow-colored product at 412 nm. Given its ability to inhibit pyrogallol autoxidation, SOD activity was determined following the methods of Marklund and Marklund^23^.

### Determination of cardiac TNF-α, Nrf-2 and HO-1 levels

Cardiac levels of TNF-α, Nrf-2 and HO-1 were determined via enzyme-linked immunosorbent assay kits obtained from eBioscience, Inc. (USA). Cytokine levels were determined according to the manufacturer’s instructions^12,24^.

### Flow cytometric analysis of PD-L1, Bcl-2, Bax and Il-6 expression

The expression values of PD-L1, Bcl-2, Bax and Il-6 in cardiac cells were evaluated via flow cytometry via PE-conjugated anti-mouse PD-L1 antibodies (BD Pharmingen, San Diego, CA, USA), PE-conjugated rat anti-mouse CD274 (PD-L1), cat. no. 568085) and conjugated anti-mouse Bax monoclonal antibodies (6A7) at concentrations of 0.1 µg/mL and 0.05 µg/mL, respectively, and 10 µl of Bcl-2 (anti-Bcl-2 antibody [100/D5] (ab692) and BD Pharmingen Il-6 Clone MP5-20F3 (RUO) were added, after which the cells were resuspended in PBS containing 0.5% BSA. The heart tissue was minced and passed through a 0.2 µm nylon mesh in Tris buffer to prepare a single-cell suspension in a 25 ml beaker. One hundred microliters of the cell suspension was added to a 12 × 75 ml polystyrene tube and then incubated with 10 μl of each antibody or the cytokines for 30 min in the dark at room temperature. The cells were analysed with a BD Accuri C6 flow cytometer (BD Biosciences, Franklin Lakes, NJ, USA) via a 488 nm blue laser, a 530/30 nm filter for FITC, and a 585/40 nm filter for PE. The voltage was set at 400 V for FITC and 600 V for PE, and the compensation was adjusted according to the manufacturer’s instructions. The data were acquired and analysed via BD Accuri C6 software. The expression levels of PD-L1, Bax, Bcl-2, and Il-6 were quantified as the percentage of positive cells and the mean fluorescence intensity (MFI) within the live cell gate, which was defined by forward scatter (FSC) and side scatter (SSC) parameters^25^**.**

### Flow cytometric analysis of annexin V

Annexin V was evaluated in single-cell suspensions of heart tissue from all experimental groups via an annexin V kit (cat. No. 556547BD, *Pharmingen FITC* apoptosis). A total of 100 μl of cell suspension from each heart sample was washed with 1 ml of PBS in a centrifuge tube and spin for 5 min at 1800 rpm. The supernatant was subsequently discarded, and the cell pellet was stained with 5 μl of annexin V-FITC and 5 μl of propidium iodide (PI) in 100 μl of 1X binding buffer. The samples were kept in the dark and allowed to incubate for fifteen minutes at room temperature. After incubation, the cells were ready for analysis via an accurate C6 flow cytometer (Becton Dickinson). The percentage of cells analysed by Accuri C6 software t represents the quadrant dot plot to perform the four phases of viable, early and late apoptosis and finally necrosis in each group^26^.

### Histopathological analysis

Heart specimens were harvested from all the experimental groups, fixed in 10% neutral buffered formalin, dehydrated by ascending grades of ethyl alcohol, cleared in xylene and embedded in molted paraffin. Four- to five-micron-thick sections were prepared and stained with hematoxylin and eosin (H&E)^27^ for histopathological examination, inspected blindly by a pathologist under a light microscope (BX43, Olympus) and photographed via CellSens dimension software (Olympus) connected to an Olympus DP27 camera.

### Evaluation of the ultrastructural picture of the heart

Heart samples were prepared for examination via electron microscopy and immersed in 2.5% glutaraldehyde in phosphate buffer. After that, the samples were washed with phosphate buffer, fixed in 1% osmium tetroxide, dehydrated with ascending ethanol scales (100%, 95%, and 70%), embedded in ebox resin capsules, stained with toluidine blue and examined via light microscopy to localize the selected area. Then, we cut ultrathin sections with a diamond knife on copper grids and stained them with uranyl acetate and then lead citrate. Finally, we examined the grids and photographed them with a JEOL-JEM-100 SX electron microscope (Japan) at 80 kilovol (Jeol Ltd., Tokyo, Japan) at the electron microscope unit of the Faculty of Agriculture, Mansoura University^28^.

### Statistical analysis

The results are reported as the means ± SD (8 rats). We used one-way analysis of variance (ANOVA) with Tukey’s post hoc test between the groups. Prior to performing ANOVA, all samples were subjected to normality testing via the Shapiro‒Wilk test. The results were statistically significant (p values < 0.05). GraphPad Prism v. 8.0 software developed by GraphPad Software, Inc., USA, was used to analyse the data.

## Results

### Effect of cordy cepin on changes in body weight, heart weight and heart-to-body weight ratio

**Fig. 1:**
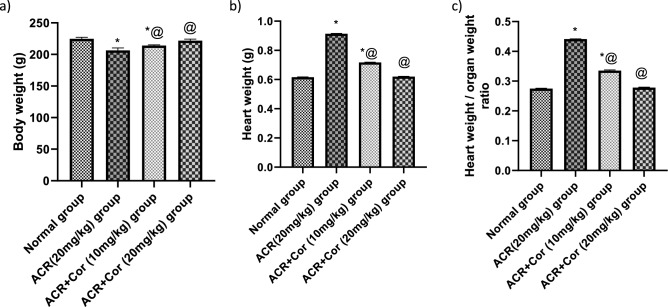
Effect of cordycepin on the changes in body weight, heart weight and heart to body weight ratio. Rats were received cordycepin (10 and 20 mg/kg) concurrently with ACR (20mg/kg) orally for 27 days. Body weight, heart weight and consequently heart to body weight ratio were calculated. Results are expressed as mean ±SD (n=8). ^*^ Significant difference from normal group *p <0.05*. ^@^ Significant difference from ACR group *p <0.05*.

Compared with the normal group, the rats that received ACR presented a decrease in body weight of nearly 91% and an increase in heart weight and heart-to-body weight ratio, reaching about, 148% and 157%, respectively (p < 0.05). Compared with those in the ACR group, the body weights of ACR model rats treated with Cor (10 mg/kg) increased, reaching about 103%, and the heart weights, heart weights and heart-to-body weight ratio, reaching approximately 78% and 77%, respectively, of those in the ACR group. Compared with the ACR rats, the ACR rats that received Cor (20 mg/kg) presented an increase in body weight, reaching approximately 107%, and a decrease in heart weight, heart weight and heart-to-body weight ratio, reaching about, 67% and 63%, respectively, compared with those of the ACR group *(p* < *0.05)* (Fig. 1).

### Evaluation of serum levels of cTnI, CK-MB and LDH

**Fig. 2:**
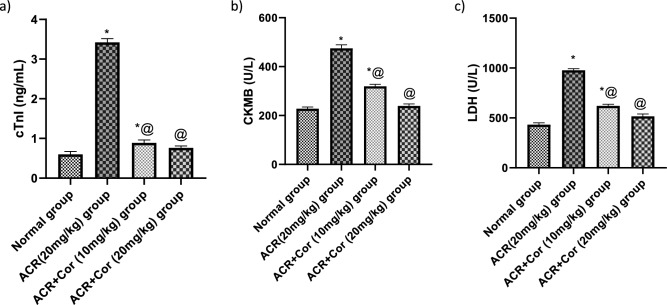
Effect of cordycepin on the serum cardiac injury values in rats intoxicated with ACR induced cardiotoxicity. Rats were received cordycepin (10 and 20 mg/kg) concurrently with ACR (20mg/kg) orally for 27 days. Serum cardiac injury values were evaluated. Results are expressed as mean ±SD (n=8). ^*^ Significant difference from normal group *p <0.05*. ^@^ Significant difference from ACR group *p <0.05*.

Figure 2 shows the effects of cordycepin on the serum levels of cTnI, CK-MB, and LDH in all the tested groups. Compared with those of the normal group, the ACR-treated groups presented significant increases in all the tested parameters, reaching approximately 5.8-fold, twofold and 2.3-fold increases, respectively. In contrast, the treatment groups that received Cor (10 mg/kg) in combination with ACR presented decreases in the tested parameters, reaching about 25%, 67% and 63%, respectively. In a dose-dependent manner. Additionally, the ACR rats that received Cor (20 mg/kg) in combination with ACR presented decreases in the tested parameters, reaching approximately 22%, 50% and 52%, respectively *(p* < *0.05)*.

### Evaluation of the cardiac contents of MDA, GSH and SOD

**Fig. 3:**
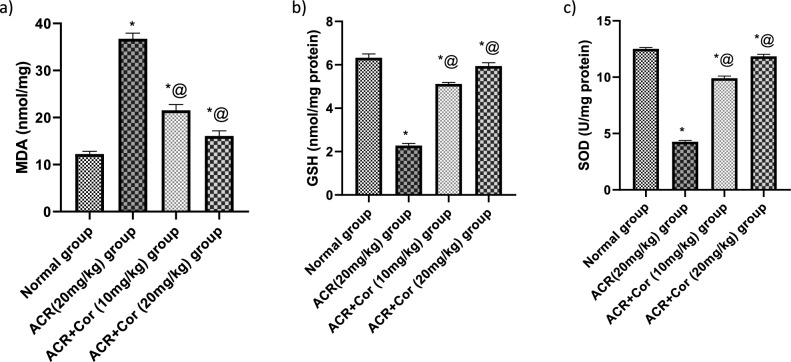
Effect of cordycepin on cardiac oxidative stress indices in rats intoxicated with ACR induced cardiotoxicity. Rats were received cordycepin (10 and 20 mg/kg) concurrently with ACR (20mg/kg) orally for 27 days. Cardiac oxidative stress indices were evaluated. Results are expressed as mean ±SD (n=8). ^*^ Significant difference from normal group *p <0.05*. ^@^ Significant difference from ACR group *p<0.05*.

Figure 3 shows a notable increase in the oxidative stress level (MDA), reaching approximately threefold greater levels, and a reduction in the levels of GSH and SOD in rats treated with ACR, reaching about 34.5% and 34.2%, respectively, compared with those in the normal group. Compared with those in the ACR group, the levels of MDA in the ACR group treated with Cor (10 mg/kg) decreased, reaching 58%, and the levels of GSH and SOD increased, reaching about, twofold and 2.3-fold, respectively. ACR-treated rats that received Cor (20 mg/kg) presented a decrease in the level of MDA, reaching about 44%, and an increase in the levels of GSH and SOD, reaching approximately 2.7- and 2.8-fold, respectively, compared with those in the ACR group *(p* < *0.05)*.

### Determination of cardiac Nrf-2, HO-1 and TNF-α level

**Fig. 4:**
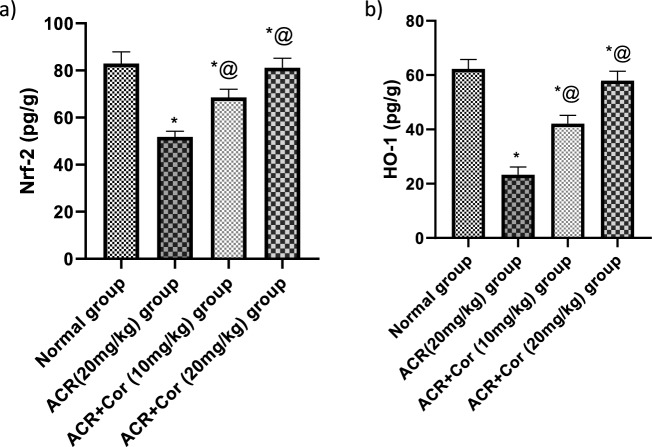
Effect of cordycepin on cardiac values of Nrf-2 and HO-1 in rats intoxicated with ACR induced cardiotoxicity. Rats were received cordycepin (10 and 20 mg/kg) concurrently with ACR (20mg/kg) orally for 27 days. Cardiac values of Nrf-2 and HO-1 were evaluated. Results are expressed as mean ±SD (n=8). ^*^ Significant difference from normal group *p <0.05*. ^@^ Significant difference from ACR group *p <0.05*.

**Fig. 5:**
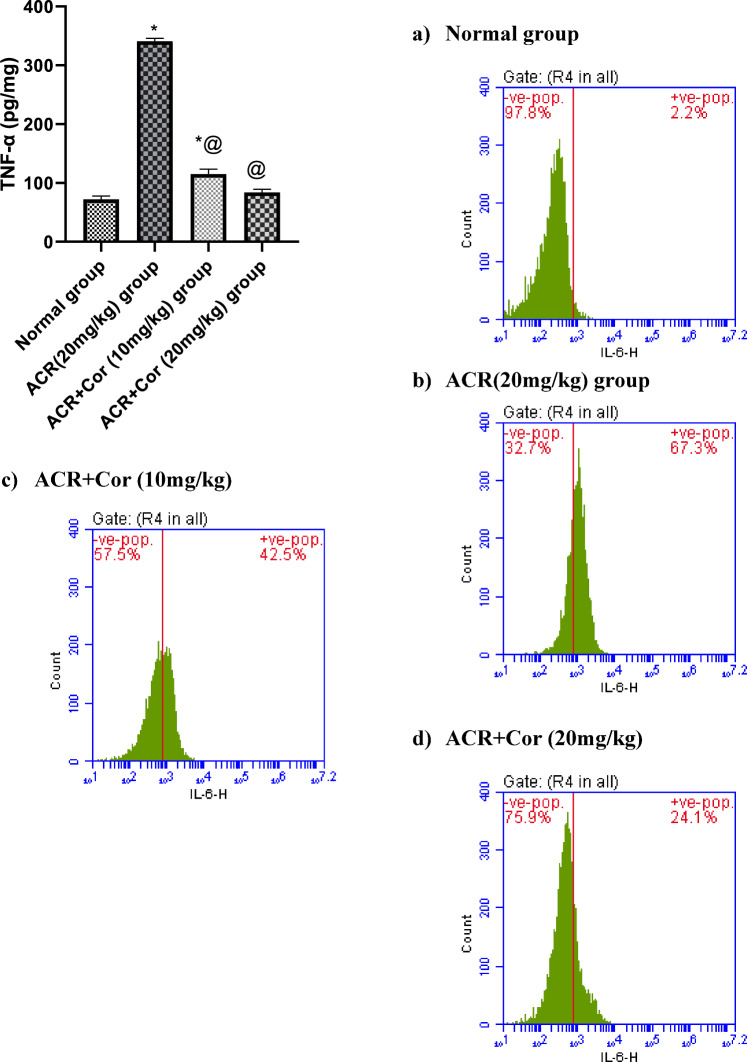
Effect of cordycepin on cardiac values of TNF-α and il-6 in rats intoxicated with ACR induced cardiotoxicity. Rats were received cordycepin (10 and 20 mg/kg) concurrently with ACR (20mg/kg) orally for 27 days. Cardiac values of TNF-α and il-6 were evaluated. Results are expressed as mean ±SD (n=8). ^*^ Significant difference from normal group *p <0.05*. ^@^ Significant difference from ACR group *p <0.05*.

The cardiac levels of Nrf-2, HO-1 and TNF-α are shown in Figs. 4,5. Compared with those in the normal group, the levels of Nrf-2 and HO-1 in the ACR-treated group were significantly reduced by 56% and 33%, respectively, and the level of TNF-α in the ACR-treated group was increased by fourfold. Compared with control rats, ACR rats treated with Cor (10 mg/kg) presented significant increases in the levels of Nrf-2 and HO-1 (200% and 146%, respectively) and a marked decrease in the level of TNF-α (33%, *p* < *0.05)*. However, the administration of Cor (10 mg/kg) succeeded in normalizing the cardiac Nrf-2, HO-1 and TNF-α levels* (p* < *0.05)*.

**Fig. 6:**
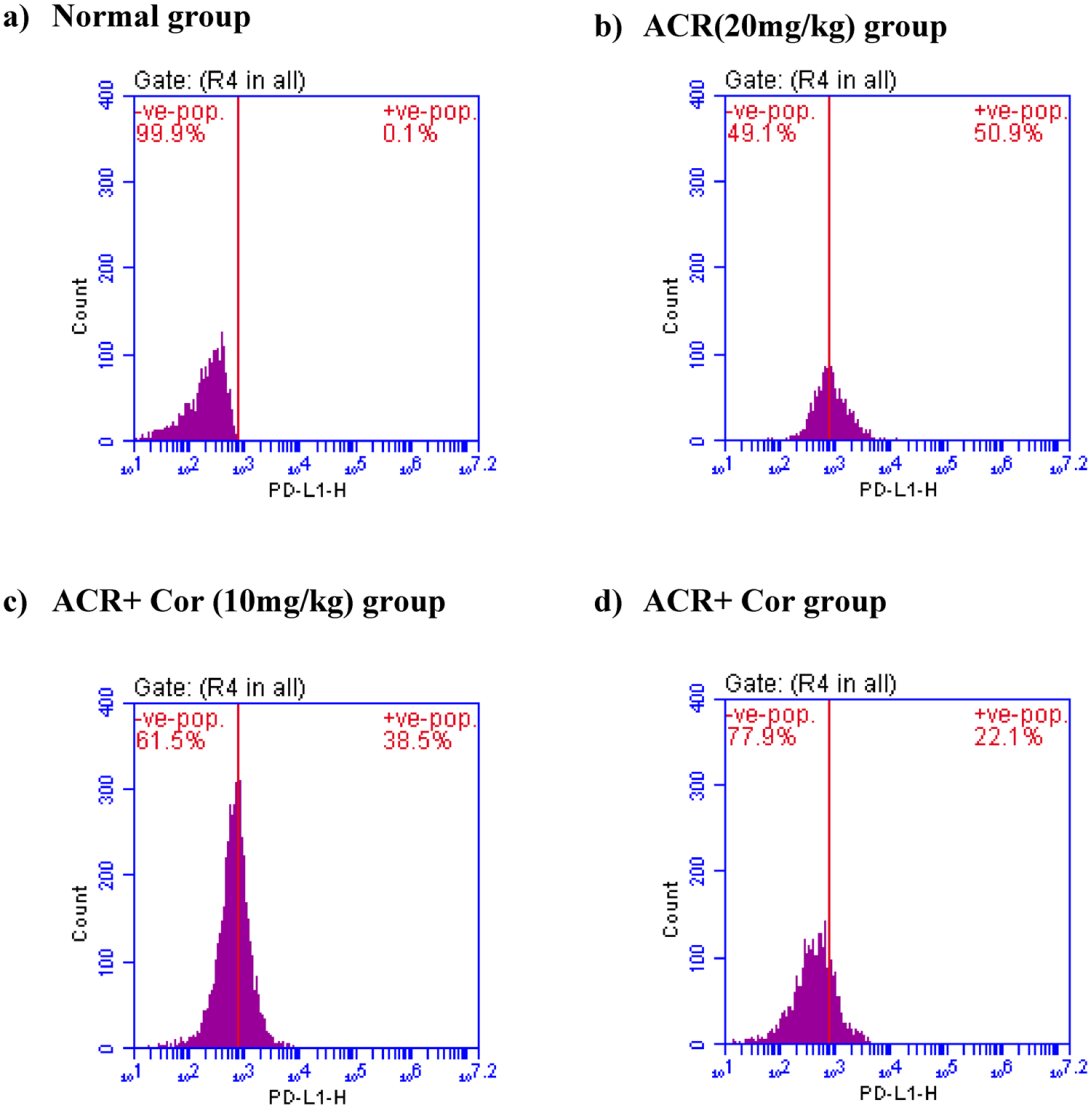
Effect of cordycepin on cardiac values of PD-L1 in rats intoxicated with ACR induced cardiotoxicity. Rats were received cordycepin (10 and 20 mg/kg) concurrently with ACR (20mg/kg) orally for 27 days. Cardiac values of PD-L1 were evaluated. Results are expressed as mean ±SD (n=8). ^*^ Significant difference from normal group *p <0.05*. ^@^ Significant difference from ACR group *p <0.05*.

### Flow cytometric analysis of IL-6, PD-L1, Bax and Bcl-2 expression

**Fig. 7:**
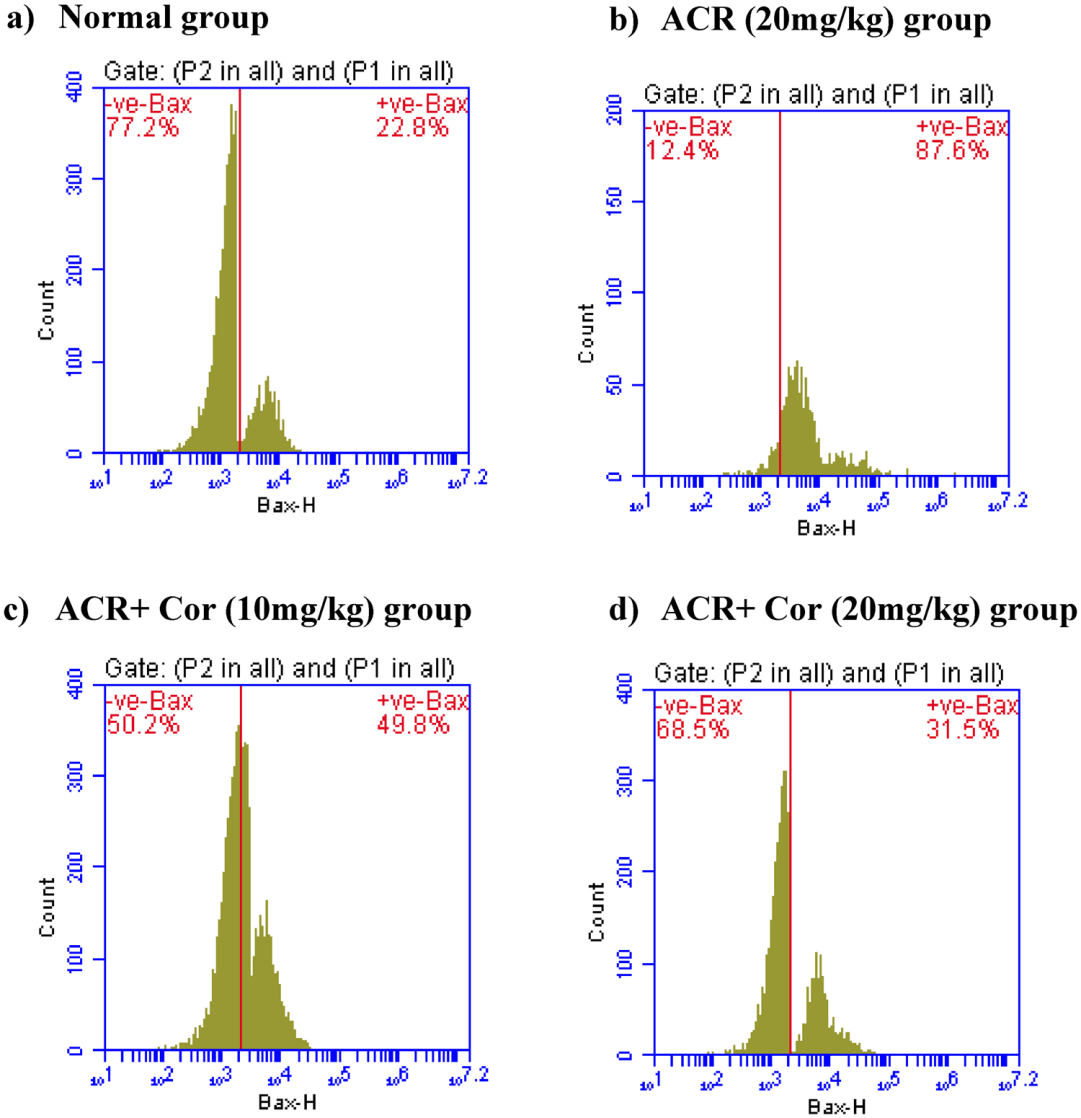
Effect of cordycepin on cardiac values of Bax in rats intoxicated with ACR induced cardiotoxicity. Rats were received cordycepin (10 and 20 mg/kg) concurrently with ACR (20mg/kg) orally for 27 days. Cardiac values of Bax were evaluated. Results are expressed as mean ±SD (n=8). ^*^ Significant difference from normal group *p <0.05*. ^@^ Significant difference from ACR group *p <0.05*.

**Fig. 8:**
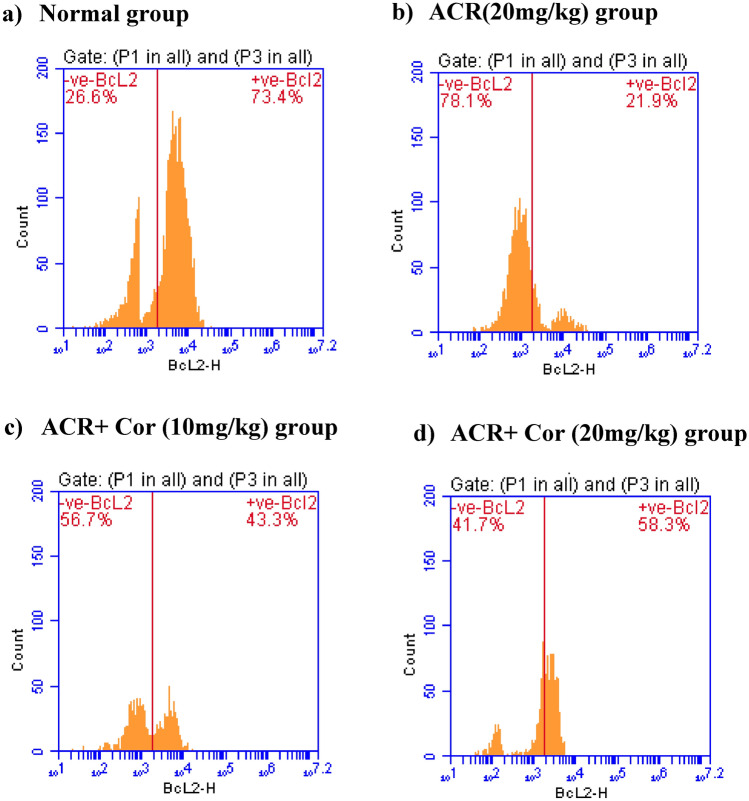
Effect of cordycepin on cardiac values of Bcl-2 in rats intoxicated with ACR induced cardiotoxicity. Rats were received cordycepin (10 and 20 mg/kg) concurrently with ACR (20mg/kg) orally for 27 days. Cardiac values of Bcl-2 were evaluated. Results are expressed as mean ±SD (n=8). ^*^ Significant difference from normal group *p <0.05*. ^@^ Significant difference from ACR group *p <0.05*.

The results of the flow cytometry analysis of IL-6, PD-L1, Bax and Bcl-2 expression are presented in the figures (5, 6, 7, 8)

The normal group 8(a) presented a normal distribution of negative (97.8%) cells with respect to Il-6 expression, whereas the intoxicated group treated with ACR (20 mg/kg) presented upregulated (67.3%) Il-6 expression in the + Ve cell population, suggesting an inflammatory response, as shown in Figure 8(b). In the ACR + Cor (10 mg/kg)-treated group, the expression of Il-6 was reduced to 42.5%, indicating amelioration of the proinflammatory response. As shown in Figure 8(d), in the group treated with ACR + Cor (20 mg/kg), Il-6 expression was reduced to 24.1% in the + Ve population.

Compared with those in the normal group, the expression levels of PD-L1 in the ACR-treated group were greater (50.9%), suggesting a cellular response to inflammation. In the groups treated with ACR** + **Cor (10 mg/kg) and (20 mg/kg), the expression levels of PD-L1 were reduced to 38.5% and 22.1%, respectively, indicating a reduced response to inflammation directly proportional to the dose of Cor.

The same results were obtained for the expression levels of Bax in the ACR-treated groups; the expression levels were elevated to 87.6% (Figure 8b), whereas in the groups treated with ACR** + **Cor (10 mg/kg) and (20 mg/kg), the levels of Bax expression decreased (49.8% and 31.5%, respectively) in a dose-dependent manner (Figures 8c, d). Bcl-2 expression levels (Fig. 8) were lower (21.9%) in the ACR-treated groups than in the normal group and then increased (43.3% and 58.3%) in both groups treated with ACR + Cor (10 or 20 mg/kg).

### Flow cytometric analysis of annexin V

**Fig. 9:**
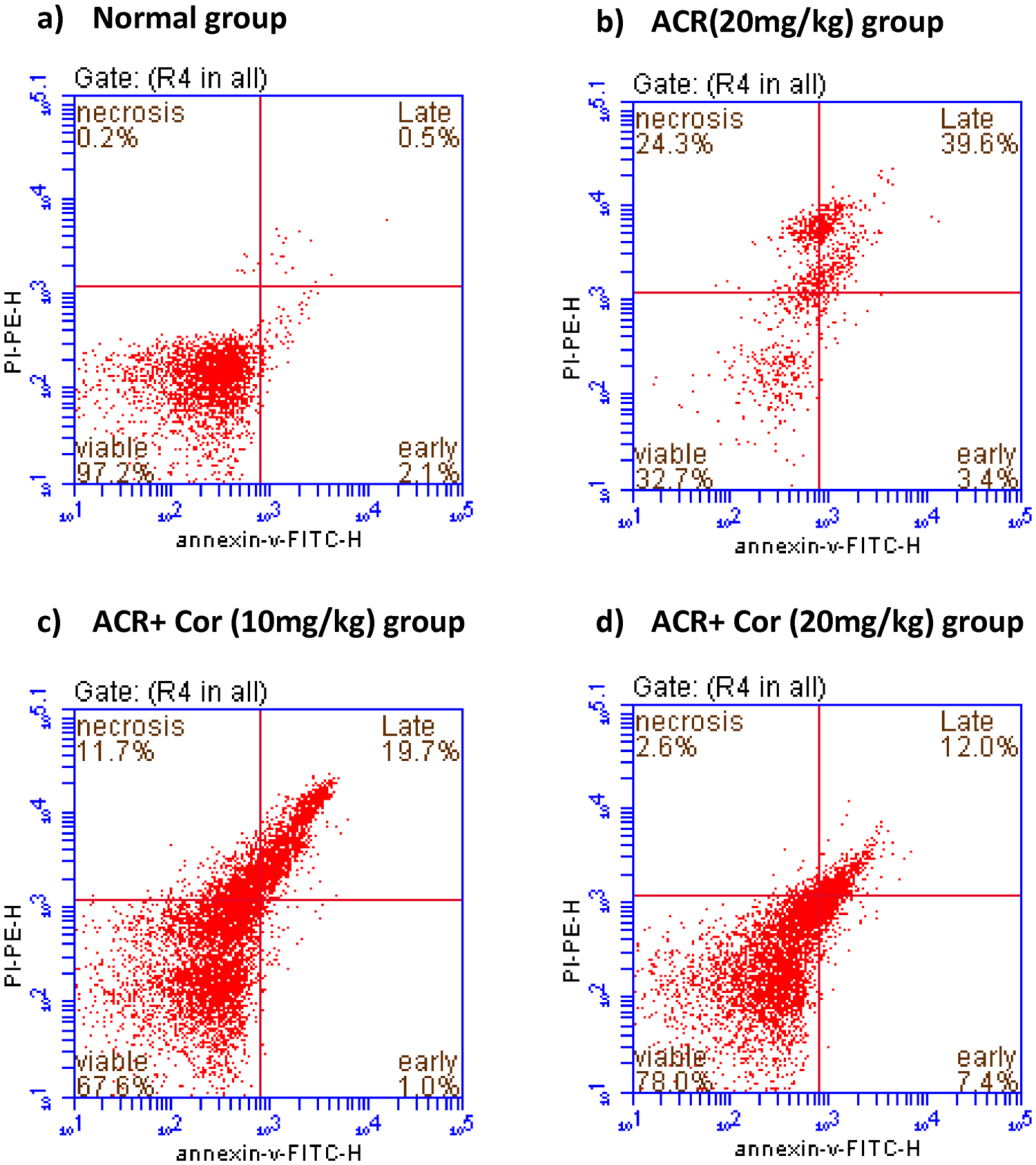
Effect of cordycepin on cardiac values of annexin V in rats intoxicated with ACR induced cardiotoxicity. Rats were received cordycepin (10 and 20 mg/kg) concurrently with ACR (20mg/kg) orally for 27 days. Cardiac values of annexin V were evaluated. Results are expressed as mean ±SD (n=8). ^*^ Significant difference from normal group *p <0.05*. ^@^ Significant difference from ACR group *p <0.05*.

Figure 9 shows normal values of viable cells (97.2%) in the lower left quadrant, which were both annexin V-negative and PI-negative. Compared with those in the normal group, the percentage of viable cells in the ACR-treated group was significantly lower (32.7%), with notable increases in the percentages of necrotic (34.3%), early apoptotic (3.4%) and late apoptotic cells (39.6%). The percentage of viable cells was greater (Figure 9c) than that in the ACR + Cor (10 mg/kg)-treated group (67.6%), and the percentage reached a maximum improvement in the ACR + Cor (20 mg/kg) (78.0%) group, as shown in Figure 9(d).

### Histopathological analysis

**Fig. 10:**
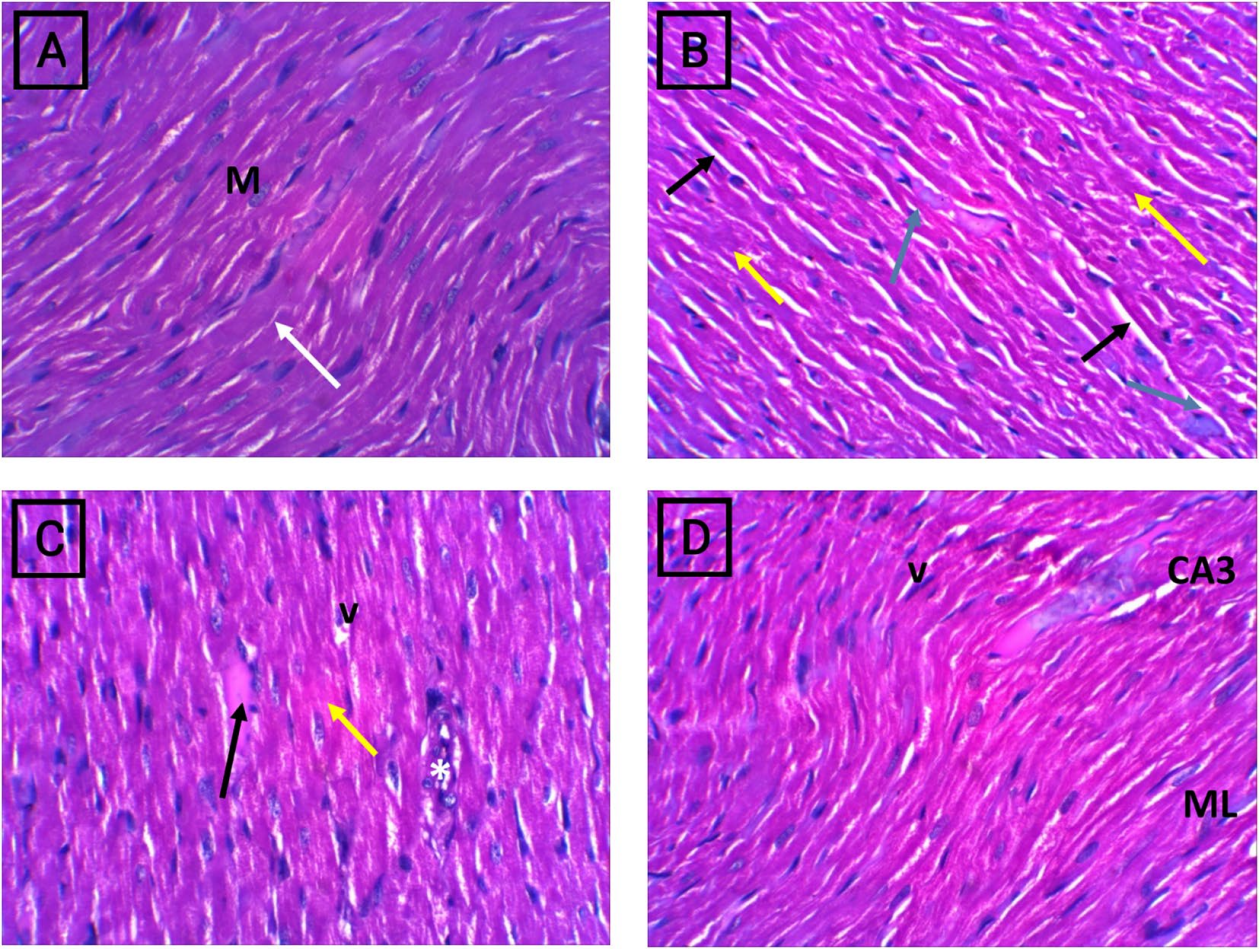
Photomicrographs of H&E staining rat heart sections of control and different experimental groups. (**A**) Normal group cardiac tissue showed intact myocardial fibers (M) that have normal staining properties and structure. (**B**) ACR group showing a varying degree of vacuolar changes (Black arrow) in the cardiac muscle fibers and increased interstitial space, fibers hyaline degeneration and necrosis (yellow arrow), acute inflammatory mononuclear cell infiltration (blue arrow). (**C**) ACR+ Cor (10mg/kg) group showing mild few wavy myocardial fibers as well as few losses of cellular constituents (black arrow), mild focal hyalinized fibers (yellow arrow) and mild cytoplasmic vacuolization (v), appear of congested capillary (*). (**D**)ACR+ Cor (20mg/kg) group showed noticed improvement in histoarchitecture with waviness of few myofibers as well as no separation of myocardial fibers. Magnification X: 400.

Light microscopic investigation of the control group revealed normal cardiac tissue histology (Fig. 10, A). An investigation of the ACR + Cor (10 mg/kg)-treated group revealed many changes compared with those in the control group. The ACR group presented varying degrees of vacuolar changes in the cardiac muscle fibres, increased interstitial space, hyaline degeneration and necrosis, and acute inflammatory mononuclear cell infiltration. (Fig. 10, B). The ACR + Cor (10 mg/kg) group presented slight cytoplasmic vacuolization, few wavy myocardial fibres, mild focal hyalinized fibres, mild cytoplasmic (v), and capillary congestion. (Fig. 10, C). The addition of Cor (20 mg/kg) to the ACR group markedly enhanced cardiac tissue organization, as did the addition of normal myofibres (Fig. 10, D).

### Evaluation of the ultrastructural picture of the heart

**Fig. 11:**
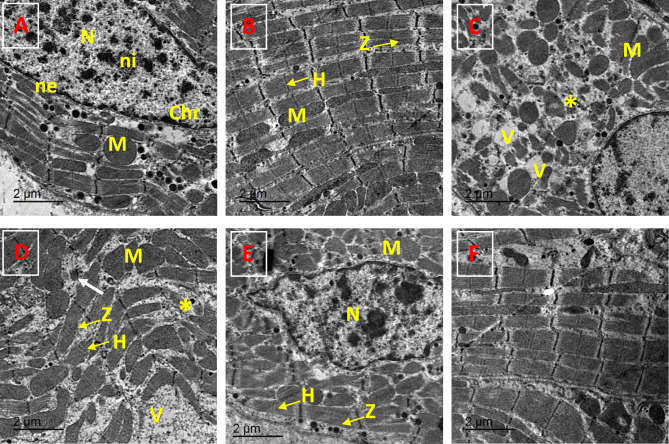
Transmission electron microscopy (TEM) of the cardiac myocytes of rat of control and experimental groups. (**A**, **B**) control group showing normal myocardial tissue with normal striated heart muscle fibers, dense mitochondria (M) and a clear nucleus (N), and muscle banding (Z and H bands). The nucleus (N) is surrounded by the nuclear envelope (ne) and contain clear chromatin masses (Chr), (**C**, **D**) ACR group revealed a marked reduction in myofibrils (*) mass with cytoplasmic degeneration, dilated sarcoplasmic reticulum and swollen mitochondria (m) and markedly abnormal in shape, fragmented gap junctions (white arrow), Fragmentation of muscle bands (Z and H) and some vacuoles (V) were also seen. (E)ACR + Cor (10mg/kg) group showing irregular nucleus (N), a regular arrangement of muscle fibers, mild swelling of myofibrillar structure with striations and swollen mitochondria. (**E**) ACR + Cor (20mg/kg) group showing ameliorative myocardial tissue with nearly normal morphology of the cardiac myocytes. Muscle Z and H bands restored it normal distribution.

Examination of heart tissue ultrastructural changes revealed that ACR-induced degenerative cytoplasmic changes included a reduction in myofibrils, dilatation of sarcoplasmic reticulum swelling of mitochondria and cytoplasmic vacuolation. In addition, fragmentation of muscle bands was also observed. In the ACR + Cor (10 mg/kg)-treated group, mild alterations, including swelling of myofibers and mitochondria, were observed; however, the muscle arrangement was normal. However, the nuclear membrane appeared irregular. The group that received ACR + Cor (20 mg/kg) exhibited marked improvement; the myocardial tissue appeared nearly normal, and the muscle Z and H bands returned to normal distributions (Fig. 11).

## Discussion

Extensive studies have shown the hazardous potential of ACR, which was first recognized as an industrial danger and was later shown to accumulate in food. Humans are exposed to ACR through many means, but mostly through diets at low concentrations over time due to increased intake of processed carbohydrates, which poses a hazard to human and animal health^29^. Although numerous studies have demonstrated that ACR is a strong carcinogen with neurotoxic properties, there have been few reports of its cardiovascular effects in animal models.

Our investigation revealed that ACR (20 mg/kg/day) caused cardiac toxicity following oral administration for 27 days. ACR-induced cardiotoxicity was characterized by a significant reduction in body weight compared with that of the normal group, which may be due to reduced feed intake or a decrease in GSH levels, which are critical for gut function and structural integrity^30^. ACR may affect thirst and hunger regulatory circuits in the hypothalamus. This finding aligns with the findings of a previous study that documented the deleterious toxic effects of ACR on body weight in mice that received an intraperitoneal injection of ACR (50 mg/kg)^31^. Co treatment with Cor, notably at a relatively high dose of 20 mg/kg, significantly increased body weight. These findings support the hypothesis that Cor may counteract ACR-induced weight loss. A previous study reported the protective role of Cor in different organ systems because of its antioxidative and anti-inflammatory properties^32,33^.

Similarly, the increase in heart weight observed in the ACR-treated group is in line with the cardiotoxic effects of ACR, which cause heart hypertrophy and stress^34^. The administration of cordycepin, particularly at relatively high doses, likely mitigated the heart enlargement induced by ACR, suggesting that cordycepin may have a protective effect by reducing oxidative stress and inflammation, mechanisms commonly implicated in cardiotoxicity. These findings are consistent with other studies suggesting the potential therapeutic effect of Cor in counteracting ACR-induced cardiac toxicity^35^.

ACR-induced cardiotoxicity was demonstrated by a significant increase in the serum levels of the cardiac biomarkers cTnI, CK-MB and LDH. cTnI is a highly specific and sensitive marker for myocardial injury^36^, and the CK-MB enzyme was identified as a measure of tissue damage in myocardial infarction and muscle disorders^37^**.** LDH is highly expressed in heart muscle and is released upon tissue damage; hence, it is employed as a marker for common injuries and diseases^38^. Previous studies have reported increases in the aforementioned cardiac indices. Our findings attributed this significant elevation to the detrimental action of ACR on diaphragmatic muscle, which causes membrane injury, loss of function and integrity of cardiac membranes and subsequent leakage of these indicators into the bloodstream.

In contrast, rats that received Cor, particularly at higher doses, demonstrated a dose-dependent reduction in these biomarkers, indicating its protective role against cardiac damage. Previous studies^20^ reported similar findings, attributing the effects of cordycepin to its antioxidative, anti-inflammatory, and antiapoptotic effects on the protection of cell membranes. However, a previous study revealed the limited effectiveness of cordycepin at lower doses, emphasizing the same observation in this study regarding the importance of dose optimization for significant therapeutic outcomes^39^. These findings support the potential of cordycepin as a cardioprotective agent against ACR-induced cardiotoxicity.

Owing to its ability to generate ROS, the cardiotoxicity of ACR could be directly attributed to oxidative stress. The present study revealed an increase in cardiac MDA values and a decrease in the levels of the antioxidant enzymes GSH and SOD, which serve as scavengers of ROS, indicating an oxidative stress status. The abovementioned disturbance reduction could be due to the effect of ACR on the cellular redox chain, which generates oxygen species, and an imbalance occurs between antioxidants and oxidation function^40^. Since oxidative stress plays a key role in ACR toxicity, the cardioprotective effect of Cor is a direct consequence of its radical-scavenging and antioxidant properties. Here, rats that received dose-dependent doses of Cor presented a remarkable reduction in cardiac MDA and increased GSH and SOD. The antioxidant activity of Cor has been reported in a previous study conducted on aged rats^41^.

To explore the molecular pathways underlying the antioxidant benefits of Cor against ACR-induced oxidative damage, Nrf2/HO-1 signaling was studied. Interestingly, Nrf2 activation and HO-1 production during cardiac toxicity are part of an adaptive response aimed at restoring redox equilibrium, lowering oxidative damage, regulating inflammation, and increasing cell survival^42^. Reports indicate that administering ACR can disrupt the Nrf2 pathway, decreasing HO-1 expression^43^. This may decrease the antioxidant defenses of cells, increasing the difficulty of resisting inflammatory assaults and oxidative stress. Our data revealed that ACR treatment resulted in a considerable decrease in renal Nrf2/HO-1 expression. Nonetheless, the administration of Cor to rats treated with ACR significantly increased Nrf2 and HO-1 expression. A previous study supported our findings, as Cor could alleviate lung inflammation induced by LPS via the activation of Nrf2 and the upregulation of HO-1 expression^44^. Overall, our findings showed that Cor reduced cardiac oxidative damage caused by ACR by altering the Nrf2/HO-1 signaling pathway.

The effects of inflammation on ACR-induced cardiac injury, as well as the underlying molecular mechanism, were subsequently studied. ACR-induced production of the inflammatory cytokines TNF-α and Il-6 has been reported in experimental studies, and our results are in accordance with these reports^45^. In our study, ACR increased the expression of these inflammatory cytokines, and treatment with Cor increased the levels of these cytokines. Our findings support the findings of a previous study in which Cor was shown to reduce the release of TNF-α and Il-6 in human bladder cancer cell lines^46^. The reduced levels of inflammatory cytokines confirmed the anti-inflammatory activity of Cor.

As previously demonstrated, ACR exposure is strongly related to cardiac damage caused by oxidative stress and apoptosis^47^. Furthermore, Nrf2 suppression induces apoptosis by decreasing the activity of antiapoptotic-related Bcl-2 and increasing the expression of proapoptotic-related Bax and programmed death ligand-1 (PD-L1)^48^. Annexin V staining is a frequently used method for detecting apoptotic cells^49^. Our data revealed that the annexin V values were greater in the ACR group. Previous evidence verified our findings and demonstrated the occurrence of cardiac apoptosis in Swiss Albino male mice intoxicated with ACR^50^. In contrast, administering Cor in a dose-dependent manner was related to considerable decreases in Bax, PD-L1 and annexin V expression, indicating that the antiapoptotic action of Cor. Moreover, Cor significantly increased the cardiac expression of Bcl-2. A previous study revealed the antiapoptotic role of Cor in rats with rotenone-induced Parkinson’s disease^51^.

Our findings were also supported by histological and ultrastructural evidence, where rats subjected to ACR presented significant histological and ultrastructural abnormalities, including cardiac degeneration, inflammatory cell infiltration, vascular congestion, and edema. On the other hand, the administration of Cor, especially 20 mg/kg, markedly improved the myocardial tissue to nearly normal levels and restored its normal distribution.

In conclusion, ACR-induced cardiotoxicity is associated with the disruption of Nrf2/HO-1 and Bax/Bcl2signaling. This is coupled with biochemical imbalance. However, Cor (20 mg/kg) confers cardiac protection by preventing ACR-induced Nrf2/HO-1 and Bax/Bcl-2 signaling dysfunction and has significant antiapoptotic effects, thus preserving cardiac function. These findings revealed that Cor (20 mg/kg) might be a potential therapeutic candidate for preventing ACR-induced cardiac toxicity.

## Data Availability

The datasets used and/or analysed during the current study available from the corresponding author on reason-able request.
